# Platinum Palladium Bimetallic Nanozymes Stabilized with Vancomycin for the Sensitive Colorimetric Determination of L-cysteine

**DOI:** 10.3390/biom13081254

**Published:** 2023-08-16

**Authors:** Han Zhao, Kai Liu, Lijie Zhou, Tingting Zhang, Zengsheng Han, Longgang Wang, Xianbing Ji, Yanshuai Cui, Jie Hu, Guanglong Ma

**Affiliations:** 1 Hebei Key Laboratory of Nano-Biotechnology, College of Environmental and Chemical Engineering, Yanshan University, Qinhuangdao 066004, China; han0325@stumail.ysu.edu.cn (H.Z.); d202210477@xs.ustb.edu.cn (K.L.); ljzhou@stumail.ysu.edu.cn (L.Z.); ttz@stumail.ysu.edu.cn (T.Z.); hujie@ysu.edu.cn (J.H.); 2 State Key Laboratory of Metastable Materials Science and Technology, Yanshan University, Qinhuangdao 066004, China; 3 Department of Environmental Engineering, Hebei University of Environmental Engineering, Qinhuangdao 066102, China; jixianbing@hebuee.edu.cn (X.J.); cuiyanshuai@hebuee.edu.cn (Y.C.); 4 Centre for Cancer Immunology, Faculty of Medicine, University of Southampton, Southampton SO16 6YD, UK; gm1c21@soton.ac.uk

**Keywords:** bimetallic, nanozymes, colorimetric, L-cysteine, detection

## Abstract

Many diseases in the human body are related to the level of L-cysteine. Therefore, it is crucial to establish an efficient, simple and sensitive platform for L-cysteine detection. In this work, we synthesized platinum palladium bimetallic nanoparticles (Van-Pt_*m*_/Pd_*n*_ NPs) using vancomycin hydrochloride (Van) as a stabilizer, which exhibited high oxidase-like catalytic activity. In addition, the catalytic kinetics of the Van-Pt_1_/Pd_1_ NPs followed the typical Michaelis–Menten equation, exhibiting a strong affinity for 3,3′,5,5′-tetramethylbenzidine substrates. More importantly, we developed a simple and effective strategy for the sensitive colorimetric detection of L-cysteine using biocompatible Van-Pt_1_/Pd_1_ NPs. The detection limit was low, at 0.07 μM, which was lower than the values for many previously reported enzyme-like detection systems. The colorimetric method of the L-cysteine assay had good selectivity. The established method for the detection of L-cysteine showed promise for biomedical analysis.

## 1. Introduction

L-cysteine is one of the sulfur-containing α-amino acids with good water solubility. Meanwhile, L-cysteine is involved in the reduction process of cells and phospholipid metabolism in the liver. The intracellular concentration of L-cysteine is usually around 30–200 μM [1]. High or low levels of L-cysteine in the body can cause diseases [2]. Therefore, it is important to establish an efficient, reliable and sensitive L-cysteine assay platform with which to detect its concentration. Currently, many methods have been reported for the detection of L-cysteine, including electrochemical methods, high-performance liquid chromatography, spin photometric methods, fluorescence detection and colorimetric detection [3,4,5,6]. However, many detection methods are limited in terms of their wide application by their cost, detection time, toxicity and environmental hazards.

In recent years, colorimetric detection has been considered as a promising method for the detection of L-cysteine due to its simplicity of operation and good visualization [7]. Many natural enzymes have been widely used in colorimetric assays. For example, Huang et al. [8] used the peroxidase activity of fig protease for the colorimetric assay of L-cysteine. The application of natural enzymes was limited due to shortcomings such as their complex extraction process, fallibility and cost.

More recently, artificial enzymes with enzyme-like activities have been developed. Since the first study of Fe_3_O_4_ nanomaterials for peroxidase-like activity in 2007 [9], nanozyme-based colorimetric sensing platforms have contributed to the rapid development of the diagnostic and bioanalytical fields. A variety of nanozymes including noble metals [10], metal oxides [11,12], carbon-based materials [13,14] and other nanomaterials [15,16,17,18] have been reported. Pandey et al. [19] reported the structural characterization of noble metal monometallic, bimetallic and trimetallic nanoparticles, in addition to evaluating their biocatalytic activity for the non-enzymatic sensing of glucose. In particular, artificial nanozymes composed of noble metal nanoparticles have a wide range of applications due to their high interfacial stability, easy preparation and modification [20], as in the case of Pt nanotubes [21], gold nanoparticles [22] and Pd nanoparticles. Compared with monometallic nanoparticles, bimetallic nanoparticles with synergistic effects have received widespread attention because of their higher catalytic activity [23,24]. Jang et al. [25] reported a TiO_2_-loaded Pt-Pd bimetallic model catalyst. Compared with the monometallic catalyst, the d electrons of Pt-Pd bimetallic nanoparticles were transferred from Pt 5d to Pd 4d upon alloying and the orbital hybridization and electronic state broadening of Pt and Pd. This led to a significant improvement in the catalytic performance of the bimetallic Pt-Pd catalyst. The metals Pt and Pd are both face-centered, cubic-structured metals with similar lattice constants; thus, they are more likely to form a homogeneous alloy. When Pt and Pd form an alloy, the coupling between the metals can improve their catalytic performance. Jin et al. [26] reported a Pd-Pt bimetallic alloy nanowire that exhibited excellent oxidase activity in an acidic environment.

Despite their small size, noble metal nanoparticles have a large specific surface area and high catalytic activity. But they tend to aggregate easily in solutions, leading to reduced activity. Therefore, the introduction of various carriers to stabilize small nanoparticles is considered one of the most effective ways to improve the catalytic activity and stability of enzyme mimics [27]. Among the nanoparticles, the carriers usually serve as a backbone to widely disperse and stabilize the active components, such as inorganic mesoporous silica [28], polymeric carriers [29] and polysaccharides, etc. [30]. Because of their good biocompatibility and easy modification, peptides have become a good means of modification on the surface of nanomaterials [31]. Vancomycin hydrochloride (Van), as an antibacterial peptide, is a glycopeptide antibiotic with a molecular weight of 1486 and has great potential for stabilizing precious metal nanoparticles.

In this study, we synthesized platinum palladium bimetallic nanoparticles (Van-Pt_*m*_/Pd_*n*_ NPs, *m* = 1, 2; *n* = 1, 2) using Van as a biological template for the first time. The particle size of the Van-Pt_*m*_/Pd_*n*_ NPs was around 5 nm. A high catalytic activity of the Van-Pt_1_/Pd_1_ NPs was achieved by exploring the preparation method and the molar ratio of platinum and palladium. Based on the Van-Pt_1_/Pd_1_ NPs’ oxidase-like and peroxidase-like enzymatic activity, we developed a simple and effective colorimetric method for the determination of L-cysteine with a low detection limit, a wide detection range and good selectivity. Importantly, the use of Van may reduce the toxicity of noble metals, which may offer the possibility for the wide application of noble metal nanozymes.

## 2. Materials and Methods

### 2.1. Materials

Vancomycin hydrochloride (Van), potassium tetrachloroplatinate (K_2_PtCl_4_), sodium tetrachloropalladate (Na_2_PdCl_4_), sodium borohydride (NaBH_4_), 3,3′,5,5′-tetramethylbenzidine (TMB), H_2_O_2_, dopamine hydrochloride (DA·HCl), p-benzoquinone (BQ), sodium nitride (NaN_3_), isopropyl alcohol (IPA), disodium ethylenediaminetetraacetate (EDTA-2Na) and dimethyl sulfoxide (DMSO) were purchased from Aladdin (Shanghai, China). The HeLa cell is a human cervical cancer cell line. Dialysis bags (MWCO = 14,000) were purchased from Laboratories Inc. (Piscataway, NJ, USA).

### 2.2. Synthesis of Van-Pt_m_/Pd_n_ Nanoparticles

(a) A total of 73 μL of Van solution (10 mM) was added to a 2 mL polyethylene (PE) tube, and then 98 μL of K_2_PtCl_4_ solution (10 mM) was added. The solution was incubated at 25 °C at 600 rpm for 12 h. Then, 10 μL of NaBH_4_ solution (1 M, dissolved in 0.3 M NaOH solution) was added, and hydrochloric acid (1 M) was added to adjust the pH of the solution to approximately 7 after 3 h. Then, 48 μL of Na_2_PdCl_4_ solution (10 mM) was added, and 10 μL of NaBH_4_ solution (1 M) was added after 12 h. After 24 h of dialysis, the product obtained was Van-Pt_2_-Pd_1_ NPs (Pt:Pd = 2:1).
(b) The Van-Pd_1_-Pt_2_ NPs (Pt:Pd = 2:1) were prepared in a similar manner to the synthesis of Van-Pt_2_-Pd_1_ NPs. The order of the K_2_PtCl_4_ and Na_2_PdCl_4_ solutions was reversed.
(c) One-pot method: Van (73 μL, 10 mM), K_2_PtCl_4_ (98 μL, 10 mM) and Na_2_PdCl_4_ (48 μL, 10 mM) solutions were mixed in PE tubes and kept at 25 °C for 12 h. Then, 20 µL of NaBH_4_ solution (1 M, dissolved in 0.3 M NaOH solution) was added and kept at 25 °C for 12 h. The solution was dialyzed for 24 h to obtain Van-Pt_2_/Pd_1_ NPs (Pt:Pd = 2:1). We prepared Van-Pt_1_/Pd_1_ NPs in the same way (Pt: Pd = 1:1). The Pt_1_/Pd_1_ NPs were prepared in water, and the other conditions were the same as those for the Van-Pt_1_/Pd_1_ NPs.

### 2.3. Characterization of Van-Pt_m_/Pd_n_ Nanoparticles

The absorbance in the wavelength range of 200–800 nm was measured with a UV-vis spectrophotometer. The morphology was photographed using transmission electron microscopy (TEM); the crystal structure of the nanoparticles was characterized using an X-ray diffraction (XRD) meter with a diffraction ratio of 10°–90°; the elements and valence states of the nanoparticles were determined via X-ray photoelectron spectroscopy (XPS); and the hydrodynamic size and zeta potential were determined with a Zetasizer Nano-ZS90.

### 2.4. Activity of Van-Pt_1_/Pd_1_ Nanoparticles

The oxidase-like activity of the Van-Pt_1_/Pd_1_ nanoparticles was determined by measuring the oxidized TMB. A total of 200 μL of Van-Pt_1_/Pd_1_ nanoparticles (C_Pt_ = 0.45 mM) was added to a 2 mL PE tube. Then, 300 μL of 0.2 M HAc-NaAc solution (pH = 3) was added, and 1000 μL of 0.2 M HAc-NaAc solution containing 0.6 mM TMB was added. The samples were incubated in a constant-temperature mixer at 25 °C and 600 rpm for 5 min. The absorbance was measured using a UV-vis spectrophotometer. In addition, the relative activity of the Van-Pt_1_/Pd_1_ NPs was determined at different pHs (pH = 1–12) and temperatures (5–65 °C). The samples added to the PE tubes were varied according to the experimental requirements.

The peroxidase-like activity of the Van-Pt_1_/Pd_1_ nanoparticles was determined by assaying the oxidized TMB produced under hydrogen peroxide conditions. 200 μL of Van-Pt_1_/Pd_1_ nanoparticles (C_Pt_ = 0.45 mM) were added to a 2 mL PE tube. Then, 300 μL, 0.2 M of HAc-NaAc solution (pH = 3) was added; 1000 μL of 0.2 M HAc-NaAc solution containing 0.6 mM TMB was added, 100 μL, 0.03 M H_2_O_2_ solution was added. The samples were incubated in a constant temperature mixer at 25 °C and 600 rpm for 2 min. The absorbance was measured by UV-vis spectrophotometer.

### 2.5. Catalytic Kinetics of Van-Pt_1_/Pd_1_ Nanoparticles

In total, 200 μL of Van-Pt_1_/Pd_1_ nanoparticles (C_Pt_ = 0.45 mM) was added to a 2 mL PE tube. Then, we added 0.2 M of HAc-NaAc solution (pH = 3) and 0.2 M HAc-NaAc solution containing 0.6 mM TMB. The absorbance was measured using a UV-vis spectrophotometer. The amount of buffer solution was 1200–300 μL at 100 μL intervals, and the amount of buffer solution containing TMB was 100–1000 μL at 100 μL intervals. The total amount of liquid in the PE tube was 1500 μL. The affinity for the substrate and the maximum rate of the catalytic reaction during enzyme catalysis was studied using Formula (1) [32].

(1) $$v=\frac{V_{m}[S]}{K_{m}+[S]}$$

Here, *V_m_* is the maximum reaction rate; [*S*] is the substrate concentration; and *K_m_* is the Michaelis–Menten constant.

### 2.6. The Mechanism of Oxidase-Like Activity

The types of reactive oxygen species (ROS) produced during catalysis were studied by adding different ROS inhibitors to the solution. A total of 200 μL of Van-Pt_1_/Pd_1_ NPs (C_Pt_ = 0.45 mM) was added to a 2 mL PE tube. Then, 1000 μL of 0.2 M HAc-NaAc solution containing 0.6 mM TMB was added, and 200 μL of different solutions of reactive oxygen inhibitor solutions (10 mM) was added. The samples were incubated in a constant-temperature mixer at 30 °C and 600 rpm for 5 min. The absorbance was measured using a UV-vis spectrophotometer. The ROS inhibitors were p-benzoquinone (BQ), sodium nitride (NaN_3_), isopropyl alcohol (IPA) and disodium ethylenediaminetetraacetate (EDTA-2Na), respectively.

### 2.7. Detection of L-cysteine Using Van-Pt_1_/Pd_1_ Nanoparticles

A total of 50 μL of Van-Pt_1_/Pd_1_ nanozymes (C_Pt_ = 0.45 mM) was added to a 2 mL PE tube, followed by 1000 μL of 0.2 M, pH = 3 HAc-NaAc solution containing 0.6 mM TMB, and then 200 μL of aqueous L-cysteine solution at different concentrations. Then, the tube was placed in a constant-temperature reaction at 30 °C and 600 rpm for 5 min. Finally, the UV-vis spectrum was measured. The standard curve of the assay was obtained using the difference in absorbance versus concentration. The real samples were replaced with different samples with different concentrations of L-cysteine containing the spiked amount, and the recoveries were calculated according to the spiked recovery Formula (2) [33].

(2) $$recovery\;rate\%=\frac{A_{i}-A_{0}}{A_{i}^{*}-A_{0}}\times 100$$

A_i_^*^ is the theoretical absorbance, and *A_i_* is the actual absorbance.

### 2.8. Biocompatibility Test

The biocompatibility of the nanozymes was determined using the MTT method. First, cells were added to 96-well plates and incubated in a cell incubator for 24 h. Then, samples (C_samples_ = 12.5–200 µg/mL) including Van and Van-Pt_1_/Pd_1_ NPs were added to the 96-well tissue culture plates and incubated for 24 h. After that, MTT (100 µL, 500 µg/mL) was added. After 4 h, the MTT solution was removed, and DMSO solution was added. Finally, the absorbance of the 96-well plates was measured using a microplate reader.

## 3. Results and Discussion

### 3.1. Characterization of Van-Pt_m_/Pd_n_ Nanoparticles

**Figure 1:**
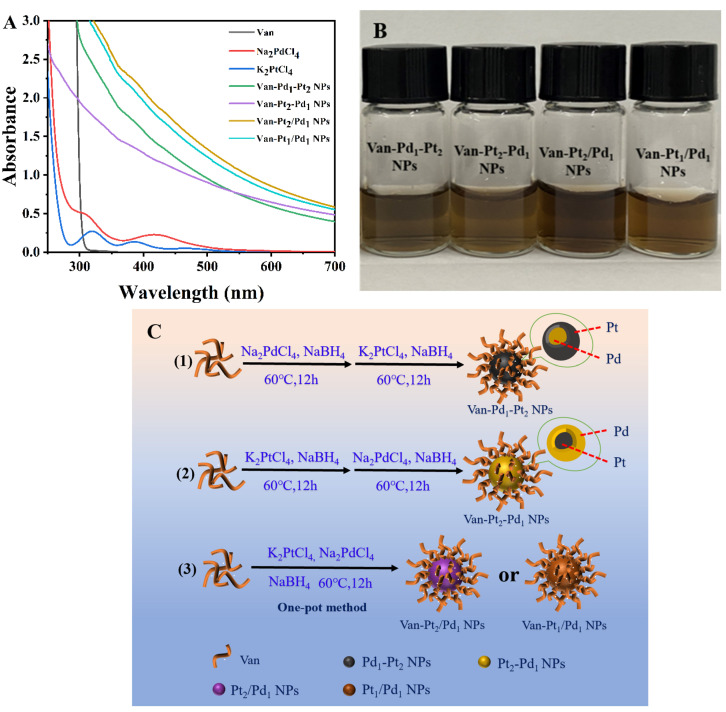
(**A**) UV-vis spectra of Van-Pt_*m*_/Pd_*n*_ NPs. (**B**) Corresponding photographs of Van-Pt_*m*_/Pd_*n*_ NPs and (**C**) schematic of the preparation of Van-Pt_*m*_/Pd_*n*_ NPs (*m* = 1, 2; *n* = 1, 2).

As shown in Figure 1A, a UV-vis spectrometer was used to scan the Van-Pt_*m*_/Pd_*n*_ nanoparticles within 250–700 nm. The characteristic bands of Pt^2+^ were at 392 nm and 329 nm, and Pd^2+^ had a distinct characteristic absorption band at 420 nm. The Van-Pt_*m*_/Pd_*n*_ NPs had no obvious characteristic absorption bands of Pt^2+^ or Pd^2+^, which may be because Pt^2+^ and Pd^2+^ were reduced to Pt and Pd, respectively. The mere absence of Pt^2+^ and Pd^2+^ transitions in the UV-vis spectra suggested that Van-Pt_*m*_/Pd_*n*_ nanoparticles may have formed. In addition, the color of the Van-Pt_*m*_/Pd_*n*_ NPs was brown (Figure 1B), similar to the color of previously reported Pt/Pd NPs [34]. All these results indicated the successful synthesis of Van-Pt_*m*_/Pd_*n*_ NPs.

**Figure 2:**
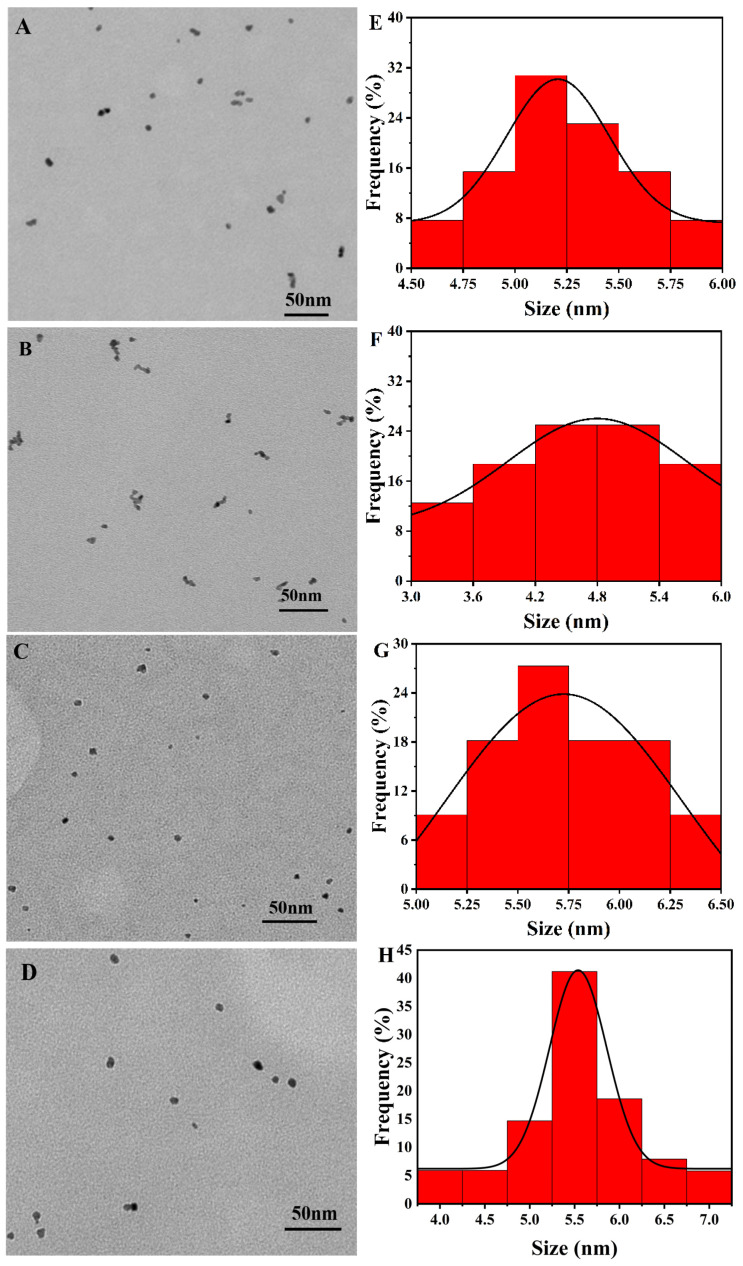
TEM images and size statistics of (**A**,**E**) Van-Pd_1_-Pt_2_ NPs, (**B**,**F**) Van-Pt_2_-Pd_1_ NPs, (**C**,**G**) Van-Pt_2_/Pd_1_ NPs and (**D**,**H**) Van-Pt_1_/Pd_1_ NPs.

As shown in Figure 2A–H, the particle sizes of the Van-Pd_1_-Pt_2_ NPs, Van-Pt_2_-Pd_1_ NPs, Van-Pt_2_/Pd_1_ NPs and Van-Pt_1_/Pd_1_ NPs were 5.3 ± 0.2 nm, 4.8 ± 0.6 nm, 5.7 ± 0.4 nm and 5.5 ± 0.5 nm, respectively. There was no significant difference in the particle size of the nanoparticles using the three synthesis methods. The Van-Pt_2_-Pd_1_ NPs and Van-Pd_1_-Pt_2_ NPs had a slight degree of aggregation. The Van-Pt_2_/Pd_1_ NPs and Van-Pt_1_/Pd_1_ NPs had better dispersion. The size of the Van-Pt_*m*_/Pd_*n*_ NPs was very small. The aggregation of the Van-Pt_2_-Pd_1_ NPs and Van-Pd_1_-Pt_2_ NPs may be due to the long reaction time.

**Figure 3:**
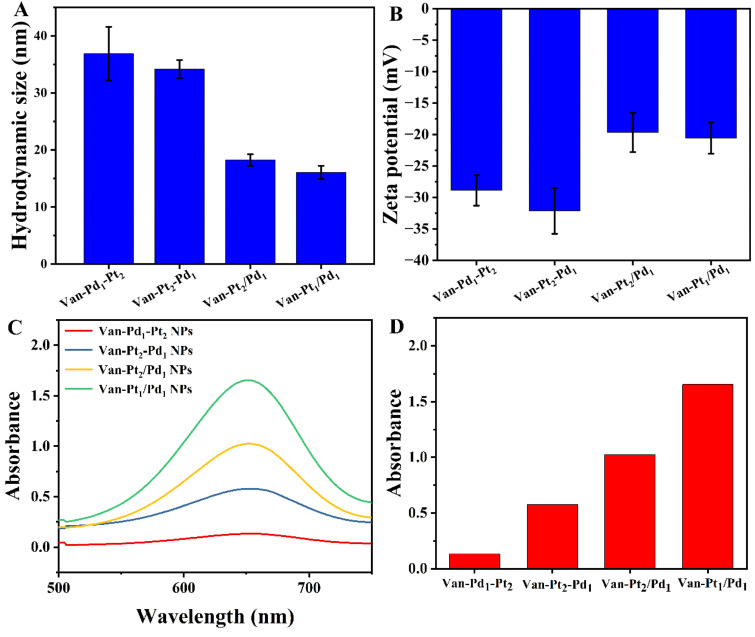
(**A**) Hydrodynamic size of Van-Pt_*m*_/Pd_*n*_ NPs. (**B**) Zeta potential of Van-Pt_*m*_/Pd_*n*_ NPs, (**C**) catalytic activities of Van-Pt_*m*_/Pd_*n*_ NPs with different synthesis methods and (**D**) absorbance of (**C**) at 652 nm, (*m* = 1, 2; *n* = 1, 2), Van-Pt_*m*_/Pd_*n*_ NPs reacted with TMB at 25 °C for 5 min.

The catalytic reaction of the nanoparticles was carried out in aqueous solutions, the hydrodynamic size and zeta potential of nanoparticles affect their catalytic activity. As shown in Figure 3A, the hydrodynamic sizes of the Van-Pd_1_-Pt_2_ NPs, Van-Pt_2_-Pd_1_ NPs, Van-Pt_2_/Pd_1_ NPs and Van-Pt_1_/Pd_1_ NPs were 36.9 ± 4.1 nm, 34.2 ± 1.6 nm, 18.2 ± 1.0 nm and 16.1 ± 1.2 nm, respectively. They were slightly larger than those observed using TEM. The reason for this is that the water molecules form a thin water film around the nanoparticles in solution, resulting in a larger hydrodynamic size than that observed with TEM [35]. Compared with the Van-Pd_1_-Pt_2_ NPs and Van-Pt_2_-Pd_1_ NPs, the Van-Pt_2_/Pd_1_ NPs and Van-Pt_1_/Pd_1_ NPs prepared using the one-pot method exhibited a smaller hydrodynamic size, which may be due to the dispersion performance of the Van-Pt_2_/Pd_1_ NPs and Van-Pt_1_/Pd_1_ NPs. In addition, the zeta potentials of the Van-Pt_*m*_/Pd_*n*_ NPs were also determined (Figure 3B). The zeta potentials of the Van-Pd_1_-Pt_2_ NPs, Van-Pt_2_-Pd_1_ NPs, Van-Pt_2_/Pd_1_ NPs and Van-Pt_1_/Pd_1_ NPs in aqueous solution were −28.5 ± 2.1 mV, −29.5 ± 3.2 mV, −18.9 ± 2.5 mV and −20.6 ± 2.5 mV, respectively. The absolute values of the zeta potentials of all four nanoparticles were greater than 18 mV. Their zeta potential favored their good stability and maintenance of catalytic activity in the solution state.

The catalytic activity of nanozymes is also closely related to their preparation methods and reaction conditions. In order to obtain Van-Pt_*m*_/Pd_*n*_ NPs with an excellent catalytic performance, we prepared nanoparticles with different metal ratios and different synthesis methods. Figure 3C shows that all the Van-Pt_*m*_/Pd_*n*_ NPs had catalytic activity. Among them, the Van-Pt_1_/Pd_1_ NPs prepared using the one-pot method exhibited the highest catalytic ability for TMB oxidation. This indicated that the nanoparticles prepared using the one-pot method had good catalytic activity. In this case, the reaction time of the Van-Pt_1_/Pd_1_ NPs prepared using the one-pot method was 12 h, while the reaction time of the Van-Pt_1_-Pd_1_ NPs and Van-Pd_1_-Pt_1_ NPs prepared in a stepwise manner was 24 h, which caused the nanoparticles to become aggregated, leading to a decrease in the activity of the oxidase-like nanoparticles as compared to those obtained using the one-pot method. Meanwhile, we also compared other metal molar ratios of Van-Pt_*m*_/Pd_*n*_ nanoparticles, i.e., *m*:*n* = 1:1, 1:2, 2:1, 1:5, 5:1, 1:10 and 10:1, as shown in Figure S1. The results showed that the Van-Pt_1_/Pd_1_ NPs had the highest catalytic activity for TMB.

In short, among the three synthesis methods, the nanoparticles synthesized using the one-pot method had better dispersion and a smaller hydrodynamic size, which caused the nanoparticles to have more active sites; hence, the one-pot method was chosen to prepare the Pt Pd bimetallic nanoparticles. When comparing the catalytic activities of the nanozymes prepared with different molar ratios of Pt to Pd, the Van-Pt_1_/Pd_1_ NPs showed the highest catalytic activity; thus, we chose the Van-Pt_1_/Pd_1_ NPs for the subsequent experiments.

**Figure 4:**
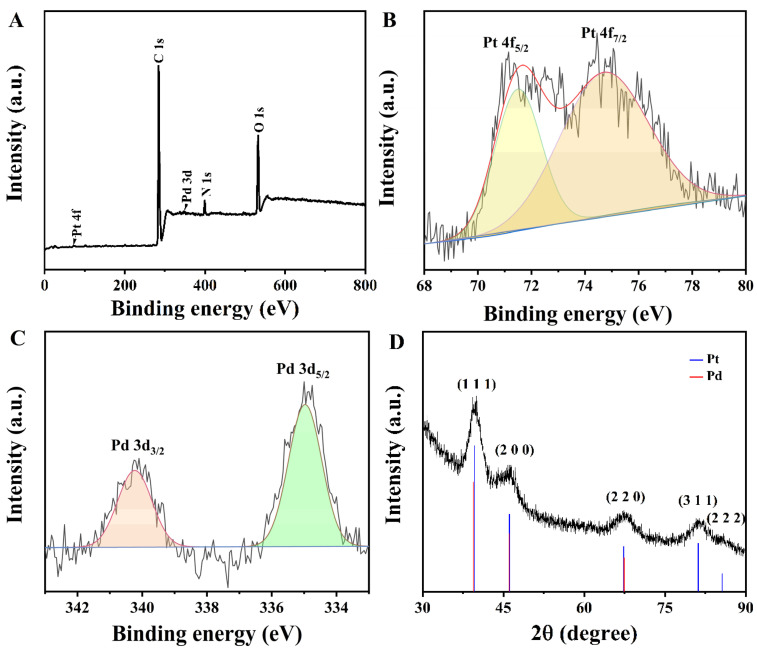
(**A**) XPS spectrum of Van-Pt_1_/Pd_1_ NPs, high-resolution XPS spectra of (**B**) Pt and (**C**) Pd, and (**D**) XRD spectrum of Van-Pt_1_/Pd_1_ NPs.

To further test our successful synthesis of Van-Pt_*m*_/Pd_*n*_ NPs, we performed XPS and XRD characterizations of the Van-Pt_1_/Pd_1_ NPs. The XPS spectra of the Van-Pt_1_/Pd_1_ NPs showed five elements, C, N, O, Pt and Pd, as demonstrated in Figure 4A. Three elements, C, N and O, were derived from the biological template of Van, while Pt and Pd elements were reduced from K_2_PtCl_4_ and Na_2_PdCl_4_, respectively. The binding energies at 71.2 eV and 74.7 eV corresponded to the Pt 4f_7/2_ and Pt 4f_5/2_ orbitals of the Pt elements in Figure 4B, respectively [36]. This binding energy coincided with the binding energy of the 4f orbital of the Pt atom, which indicated that the Pt^2+^ in K_2_PtCl_4_ had been reduced to a Pt atom. In addition, the binding energies in Figure S2 are 284.6 eV, 286.2 eV and 288.6 eV, for which the corresponding chemical groups are C-C, C-O and C=O, respectively, and the C element was provided by Van [37,38,39]. Figure 4C shows the XPS spectrum of Pd 3d. The binding energies of 335.0 eV and 340.5 eV correspond to Pd 3d_5/2_ and Pd 3d_3/2_ orbitals, respectively, which were consistent with the 3d orbital binding energy of Pd at a valency of 0 in the Pt/Pd alloy [40]. Therefore, the successful loading of Pt/Pd alloy nanoparticles on the template of Van could be determined using XPS spectra, further proving our successful synthesis of Van-Pt_1_/Pd_1_ NPs. The XRD results showed that the diffraction peaks appeared at 39.76°, 46.24°, 67.45°, 81.28° and 85.71°, which correspond to the (1 1 1), (2 0 0), (2 2 0), (3 1 1) and (2 2 2) crystal planes of Pd and Pt, respectively (Figure 4D). Among the peaks, 39.76° and 46.24° are attributed to the planar crystal structure of Pt and Pd nanoparticles. Comparing the reference code 01-001-1194 for Pt and the reference pair 46–1043 for Pd, the diffraction peak is slightly higher than that of Pt and slightly lower than that of Pd. It is clear that the diffraction angles of the Van-Pt_1_/Pd_1_ NP alloy are in the middle of the diffraction peaks of Pt and Pd [41]. Thus, the XRD of the Pt-Pd alloy nanoparticles proved that we successfully synthesized Van-Pt_1_/Pd_1_ NPs using Van. The other peaks observed at 67.45°, 81.28° and 85.71° were related to the planar formation of Pt and Pd in the Van-Pt_1_/Pd_1_ NPs.

### 3.2. Catalytic Activity of Van-Pt_1/_Pd_1_ NPs

**Figure 5:**
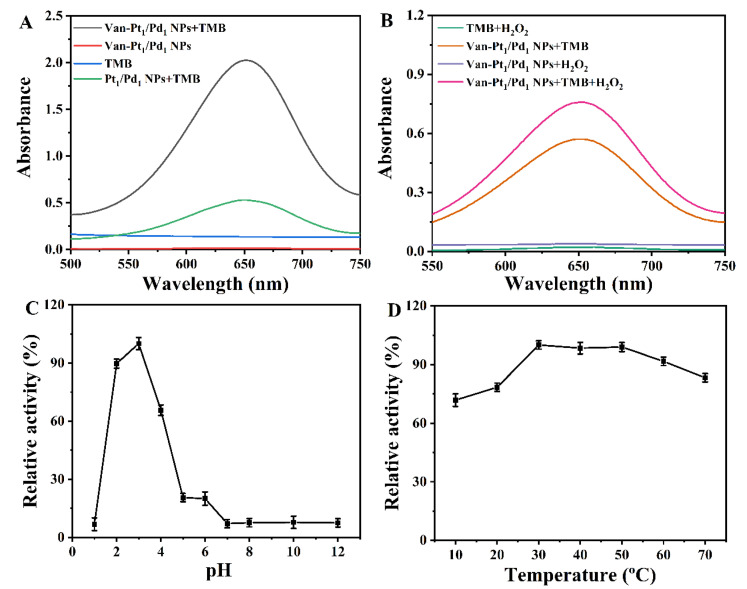
(**A**) Oxidase-like activity of Van-Pt_1_/Pd_1_ NPs with a reaction time of 5 min at 25 °C. (**B**) Peroxidase-like activity of Van-Pt_1_/Pd_1_ NPs with a reaction time of 2 min at 25 °C. (**C**) Optimal pH and (**D**) optimal temperature of Van-Pt_1_/Pd_1_ NPs.

To test the catalytic activity of the Van-Pt_1_/Pd_1_ NPs, we designed the following groups: 1TMB, 2Van-Pt_1_/Pd_1_ NPs, 3TMB + Van-Pt_1_/Pd_1_ NPs and 4TMB+ Pt_1_/Pd_1_ NPs. As shown in Figure 5A, the TMB + Van-Pt_1_/Pd_1_ NP group showed the highest characteristic absorption peak, and this characteristic absorption peak was provided by the oxidized TMB (oxTMB) [42,43]. During this experiment, the absorbance of the TMB and Van-Pt_1_/Pd_1_ NPs at 652 nm was close to 0, while the absorbance of the TMB + Pt_1_/Pd_1_ NP group was only 26% that of the TMB + Van-Pt_1_/Pd_1_ NPs group. Therefore, the oxidase-like activity originated from the synthesized Van-Pt_1_/Pd_1_ NPs.

We also designed different groups to investigate the peroxidase-like activity of Van-Pt_1_/Pd_1_ NPs: 1TMB + H_2_O_2_, 2Van-Pt_1_/Pd_1_ NPs + H_2_O_2_, 3TMB + Van-Pt_1_/Pd_1_ NPs and 4TMB + Van-Pt_1_/Pd_1_ NPs + H_2_O_2_. As shown in Figure 5B, the characteristic absorption peak of oxTMB at 652 nm was the highest in the Van-Pt_1_/Pd_1_ NPs + TMB + H_2_O_2_ group after 2 min of reaction. In addition, the TMB + H_2_O_2_ and Van-Pt_1_/Pd_1_ NPs + H_2_O_2_ groups did not show the characteristic absorption peak of oxTMB at 652 nm. Importantly, oxTMB peaks were observed in the TMB + Van-Pt_1_/Pd_1_ NP group under the influence of Van-Pt_1_/Pd_1_ NPs oxidase-like activity, but the absorbance was 25% lower than that of the TMB + Van-Pt_1_/Pd_1_ NPs + H_2_O_2_ group. Therefore, the Van-Pt_1_/Pd_1_ NPs had not only good oxidase-like activity but also peroxidase-like activity. The oxidase-like activity of the Van-Pt_1_/Pd_1_ NPs was very high; thus, the oxidase-like activity of the Van-Pt_1_/Pd_1_ NPs was investigated in the subsequent experiments.

The oxidase-like activity of Van-Pt_1_/Pd_1_ NPs is influenced by external conditions, the main influencing factors being pH and temperature. In order to find the optimal conditions for enzyme catalysis, the oxidase-like activity of the Van-Pt_1_/Pd_1_ NPs at different pHs and temperatures was investigated. From Figure 5C, it can be seen that the Van-Pt_1_/Pd_1_ NPs had the best oxidase-like activity at pH = 3. The oxidase-like activity of the Van-Pt_1_/Pd_1_ NPs decreased at other pHs. Therefore, the optimal pH for the oxidase-like activity of the Van-Pt_1_/Pd_1_ NPs was 3. The pH affects the binding of the TMB with the Van-Pt_1_/Pd_1_ NPs. The substrate TMB should not be suitable for binding to the Van-Pt_1_/Pd_1_ NPs at a non-optimal pH, leading to a decrease in oxidase-like activity. Furthermore, Van-Pt_1_/Pd_1_ NPs should have the highest amount of reactive oxygen species at pH = 3. The effect of temperature on the enzyme activity was then explored under the conditions of the optimal pH. In Figure 5D, it can be seen that the highest value of the nanozymes’ activity was reached at 30 °C. The activity of the nanozymes decreased at all other temperatures. However, the activity of the Van-Pt_1_/Pd_1_ NPs was maintained at a minimum of approximately 70%. Therefore, the optimal temperature for the Van-Pt_1_/Pd_1_ NPs was 30 °C, and they had a wide temperature range of catalytic performance. Thus, we can define the optimal conditions for the enzymatic activity of Van-Pt_1_/Pd_1_ NPs as pH = 3 and 30 °C. Subsequent experiments could be performed under these conditions. In addition, the catalytic activity of nanozymes is also related to nanoscale factors, such as their size, morphology and surface, which significantly affect their activity. Nayak et al. [44] assembled polyoxometalate (POM) (phosphotungstic acid (PTA)/phosphomolybdic acid (PMA)) nanoclusters and glucose oxidase (GOx) into microsphere structures, which facilitated the better diffusion of the reactants, intermediates and products due to the small size of the microspheres. This resulted in a 3–5-fold increase in the peroxidase-like activity of the PTA nanoclusters in the nanozyme microspheres.

**Figure 6:**
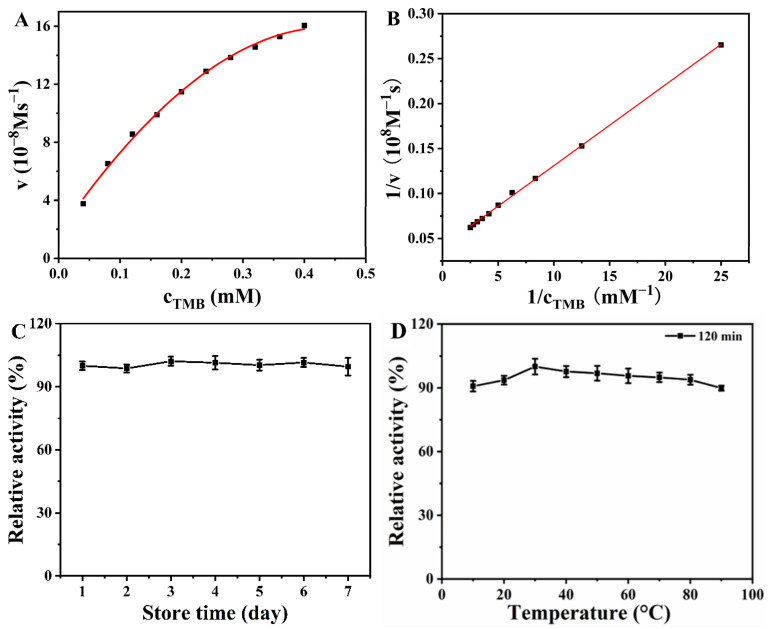
(**A**) Catalytic kinetic diagram of different TMB concentrations. (**B**) is the double-inverse curve of (**A**). (**C**) The seven-day stability of the Van-Pt_1_/Pd_1_ NPs and (**D**) the stability of the Van-Pt_1_/Pd_1_ NPs at different temperatures. Van-Pt_1_/Pd_1_ NPs were incubated at different temperature for 2 h.

To investigate the catalytic activity of the Van-Pt_1/_Pd_1_ NPs, the kinetic characterization of the nanozymes was required. The reaction kinetics of the nanozymes were determined by varying the concentration of the substrate TMB [32]. As shown in Figure 6A, the catalytic reaction followed the Michaelis–Menten equation in the concentration range of the substrate TMB (0.04–0.4 mM). As shown in Figure 6B, the standard equation was obtained as y = 0.00899x + 0.04109 (R^2^ = 0.999) using the double-inverse data in Figure 6A. The *K_m_* and *V_max_* values of the Van-Pt_1_/Pd_1_ NPs were 0.218 mM and 24.337 × 10^−8^ Ms^−1^, respectively. It can be seen from Table 1 that the *K_m_* of the Van-Pt_1_/Pd_1_ NPs was smaller compared to the other nano-enzymes, such as ZIF-67 (13.69 mM), PdPt_3_-LNT NDs (0.263 mM), CeM (0.66 mM) and Cy-AuNCs (1.925 mM). Thus, the Van-Pt_1_/Pd_1_ NPs had an excellent affinity for TMB. In addition, the Van-Pt_1_/Pd_1_ NPs had a larger *V_max_* (24.337 × 10^−8^ Ms^−1^) compared to the other nanomaterials, including the PdPt_3_-LNT NDs (2.88 × 10^−8^ Ms^−1^), Pt-HMCN (15.4 × 10^−8^ Ms^−1^), Pd_150_-PCRP NPs (15.58 × 10^−8^ Ms^−1^) and N-CQDs (4.49 × 10^−8^ Ms^−1^), which indicated that the Van-Pt_1_/Pd_1_ NPs had a better catalytic effect compared to monometallic materials and non-precious metals. Therefore, the Van-Pt_1_/Pd_1_ NPs had excellent oxidase-like activity, as shown not only by their larger *V_max_* but also by their excellent affinity with TMB.

Meanwhile, the oxidase-like activity of the Van-Pt_1_/Pd_1_ NPs was maintained at around 100% after one week of storage under ambient conditions, as shown in Figure 6C. Although the oxidase-like activity of the Van-Pt_1_/Pd_1_ NPs fluctuated to some extent, the variation was within the range of 98–102%. In addition, after 120 min of incubation in the temperature range of 10–90 °C, the catalytic performance of the Van-Pt_1_/Pd_1_ NPs remained around 90% in the range of 70–90 °C, as shown in Figure 6D. These findings indicated that the Van-Pt_1_/Pd_1_ NPs had good stability in different pH conditions and temperature tolerance, and they had good catalytic activity in extreme environments.

### 3.3. Mechanism of the Oxidase-Like Activity of Van- Pt_1_/Pd_1_ NPs

The oxidation reaction of TMB based on Van-Pt_1_/Pd_1_ NPs is closely related to reactive oxygen species [20]. Reactive oxygen species include as singlet oxygen (^1^O_2_), hydroxyl radical (·OH), superoxide anion (O_2_·^−^), etc. The mechanism of the oxidase-like activity of Van-Pt_1_/Pd_1_ NPs was investigated by adding different reactive oxygen species inhibitors, such as BQ, NaN_3_ and IPA, which have quenching effects on O_2_·^−^, ^1^O_2_ and ·OH, respectively.

**Figure 7:**
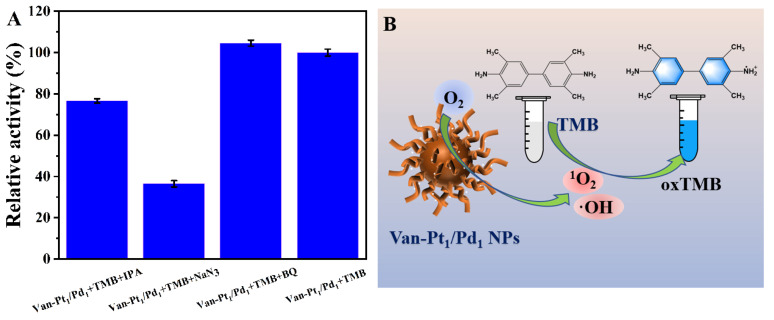
(**A**) Mechanism of oxidase-like activity and (**B**) catalytic process of Van-Pt_1_/Pd_1_ NPs. The reaction was at 30 °C, pH = 3, for 5 min. BQ, NaN_3_ and IPA had suppressive effects on O_2_^−^, ^1^O_2_ and ·OH, respectively.

To investigate the mechanism of the oxidase-like activity of Van-Pt_1_/Pd_1_ NPs, different experimental groups were set up, as shown in Figure 7A. It showed that the addition of NaN_3_ into the Van-Pt_1_/Pd_1_ NPs + TMB system (36%) had the greatest effect on the absorbance of the reaction, followed by the effect of IPA (77%), while BQ (104%) had the least effect and showed almost no difference compared with the control group of Van-Pt_1_/Pd_1_ NPs + TMB (100%). Therefore, the type of reactive oxygen species produced by the Van-Pt_1_/Pd_1_ NPs with oxidase-like activity was mainly ^1^O_2_, containing a small amount of ·OH with no O_2_·^−^ production.

### 3.4. L-cysteine Assay

**Figure 8:**
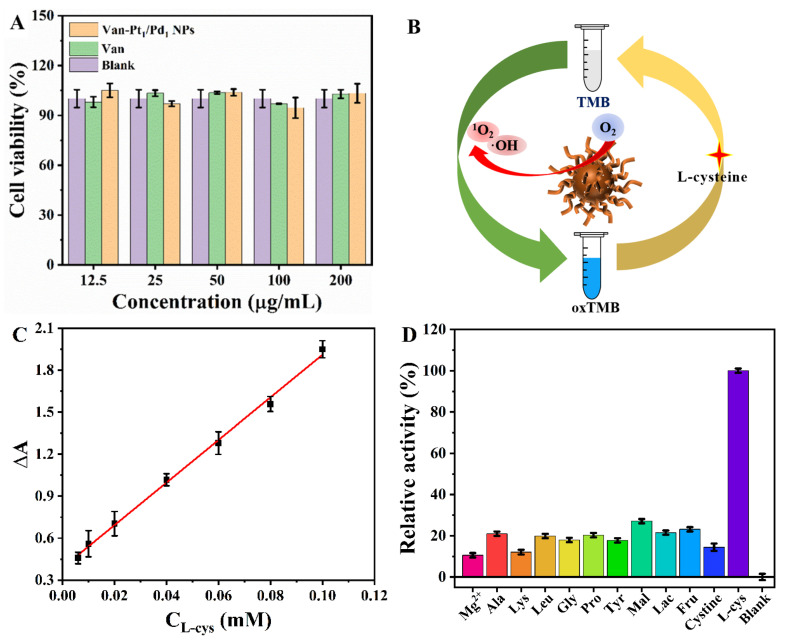
(**A**) Biocompatibility of Van-Pt_1_/Pd_1_ NPs. (**B**) Schematic diagram of Van-Pt_1_/Pd_1_ NPs for L-cysteine detection. (**C**) Linear fit of ΔA for L-cysteine in the concentration range of 6–100 μM and (**D**) selectivity of Van-Pt_1_/Pd_1_ NPs.

Here, the cytotoxicity of the Van-Pt_1_/Pd_1_ NPs and Van was explored using the MTT assay. As shown in Figure 8A, when the concentration of the Van-Pt_1_/Pd_1_ NPs and Van was 200 µg/mL, the cell viability was maintained above 90%. This indicated that the Van-Pt_1_/Pd_1_ NPs were non-cytotoxic, as compared to previously reported Pt NPs and Pd NPs [52]. Therefore, the Van-Pt_1_/Pd_1_ NPs synthesized using the bio-template method have good biocompatibility. As shown in Figure 8B, L-cysteine is a reducing biomass that can reduce oxTMB to TMB. Therefore, we could use the excellent oxidase-like activity of Van-Pt_1_/Pd_1_ NPs to establish a standard curve for the L-cysteine assay. The experimental system included Van-Pt_1_/Pd_1_ NPs, TMB and L-cysteine. The UV-vis spectrum of the solution was detected using a UV-vis spectrophotometer. As shown in Figure 8C, the calibration showed a good linear relationship with the absorbance value at 652 nm. The standard detection equation of the Van-Pt_1_/Pd_1_ NPs for L-cysteine was Y = 0.379 + 15.367 × C_L-cysteine_ (R^2^= 0.997), while the corresponding detection range of the L-cysteine concentration was 6–100 μM, and the detection limit was 0.0703 μM. Table 2 shows a comparison of the detection ranges and detection limits of L-cysteine for different materials. The Van-Pt_1_/Pd_1_ NPs (6–100 μM) had a wider linear range than the other sensors, including the MoS_2_-Au@Pt (0.8–54.4 μM), SPB@Pt NPs (0.4–3.5 μM) and Au-Ag (0.075–2 μM). In addition, Van-Pt_1_/Pd_1_ NPs had a lower detection limit (0.07 μM) than other sensors, such as PdPt_3_-LNT NDs (3.10 μM), Cu@Au/Pt (4.00 μM), VS_4_ NPs (2.50 μM) and CuMnO_2_ NFs (11.26 μM). Therefore, the colorimetric method had a wide linear detection range and a low sensitivity detection limit.

The selectivity of the Van-Pt_1_/Pd_1_ NPs was investigated by detecting L-cysteine (L-cys) and potentially interfering substances, such as Mg^2+^, alanine (Ala), phenylalanine (Phe), leucine (Leu), glycine (Gly), proline (Pro), glutamic acid (Glu), maltose (Mal), lactose (Lac) and fructose (Fru). As shown in Figure 8D, the absorbance of L-cysteine was much higher than that of the other interfering agents, even when the concentration of interfering agents was three times higher than that of L-cysteine. This indicated that the Van-Pt_1_/Pd_1_ NPs had good selectivity as probes for detecting L-cysteine. The selectivity of the Van-Pt_1_/Pd_1_ NPs for L-cysteine was high and reasonable, as compared with other reports [58,62].

To assess the potential for, and feasibility of, application in practical assays, different L-cysteine levels in mouse serum were monitored using a standard addition method. As shown in Table 3, the recoveries are 98.8% and 105.6%, respectively, indicating that the nanozymes can be applied in real samples. The good detection performance of Van-Pt_1_/Pd_1_ NPs may be related to the smaller particle size and better stability of the nanoparticles.

## 4. Conclusions

In conclusion, we successfully synthesized a Van-Pt_1_/Pd_1_ NP bimetallic nanozyme with good oxidase-like activity and peroxidase-like activity by exploring the synthesis methods and metal ratios. The catalytic kinetics of the Van-Pt_1_/Pd_1_ NPs was in accordance with the typical Michaelis–Menten equation, and the smaller *K_m_* proved that the Van-Pt_1_/Pd_1_ NPs had a good affinity for TMB. The Van-Pt_1_/Pd_1_ NPs had a good storage stability. Meanwhile, the Van-Pt_1_/Pd_1_ NPs were almost non-cytotoxic, as measured using the MTT assay. More importantly, a simple, fast and reliable L-cysteine assay was established using the prepared Van-Pt_1_/Pd_1_ NPs with a wide detection range of 6–100 μM and a low detection limit of 0.07 μM. In conclusion, stable Van-Pt_1_/Pd_1_ NPs were synthesized successfully and could be applied in the field of biomass detection.

## Figures and Tables

**Table 1:** Comparison of *K_m_* and *V_max_*.

Materials	Substrate	*K_m_* (mM)	*V_max_* ( × 10^−8^ Ms^−1^)	Reference
Van-Pt_1_/Pd_1_ NPs	TMB	0.218	24.337	this work
PdPt_3_-LNT NDs	TMB	0.263	2.88	[45]
Pt-HMCN	TMB	0.124	15.4	[46]
Pd_150_-PCRP NPs	TMB	0.2	15.58	[47]
N-CQDs	TMB	0.515	4.49	[48]
CeM	TMB	0.66	1.71	[49]
Cy-AuNCs	TMB	1.925	212.3	[50]
ZIF-67	TMB	13.69	31.96	[51]

**Table 2:** Comparison of L-cysteine detection range and detection limit for different materials.

Materials	Detection Method	Linear Range (μM)	LOD (μM)	Reference
Van-Pt_1_/Pd_1_ NPs	Colorimetry	6–100	0.07	this work
PdPt_3_-LNT NDs	Colorimetry	0–200	3.10	[45]
MoS_2_-Au@Pt	Colorimetry	0.8–54.4	0.50	[53]
SPB@Pt NPs	Colorimetry	0.4–3.5	0.11	[54]
Cu@Au/Pt	Colorimetry	0–400	4.00	[55]
VS_4_ NPs	Colorimetry	5–100	2.50	[56]
CuMnO_2_ NFs	Colorimetry	20–300	11.26	[57]
OV-Mn_3_O_4_ NFs	Colorimetry	5–800	1.31	[58]
Ag NPs	Colorimetry	0.001–1	0.001	[59]
silver NPs	Colorimetry	1.5–6	0.05	[60]
QX-AgNPs	Colorimetry	10–60	0.0027	[61]
PQDs	Fluorescence	0–800	28.11	[62]
Au-Ag	Fluorescence	0.075–2	0.04	[63]
oPAD	Electrochemical	10–800	5.50	[64]

**Table 3:** Recovery of L-cysteine assay in different samples.

Sample	Added L-cysteine Concentration (μM)	Found L-cysteine Concentration (μM)	Recovery (%)	RSD (%)
Mouse serum	50	52.1	100.2	0.57
	90	90.1	102.8	1.11

## Data Availability

The data presented in this study are available on request from the corresponding author.

## Supplementary Materials

The following supporting information can be downloaded at: https://www.mdpi.com/article/10.3390/biom13081254/s1, Figure S1: Oxidase-like activity of Van-Pt_*m*_/Pd_*n*_ NPs with different ratios of Pt and Pd at 652 nm; Figure S2: High-resolution XPS spectrum of C; Figure S3: Infrared spectra of Van and Van-Pt_1_/Pd_1_ NPs.
