# Supplementary material for: Platinum Palladium Bimetallic Nanozymes Stabilized with Vancomycin for the Sensitive Colorimetric Determination of L-cysteine
Source: Biomolecules. 2023 Aug 16;13(8):1254. doi: 10.3390/biom13081254 (PMC10452367; doi:10.3390/biom13081254)
Supplement: Supplementary file 1 [file biomolecules-13-01254-s001.zip › biomolecules-2527232-supplementary.pdf]

SUPPORTING INFORMATION

Platinum Palladium Bimetallic Nanozymes Stabilized with Vancomycin for the Sensitive Colorimetric Determination of L-cysteine

Han Zhao [1,2], Kai Liu [1], Lijie Zhou [1], Tingting Zhang [1], Zengsheng Han [1,*], Longgang Wang [1,2,*], Xianbing Ji [3], Yanshuai Cui [3], Jie Hu [1] and Guanglong Ma [4]

[1] Hebei Key Laboratory of Nano-biotechnology, College of Environmental and Chemical Engineering, Yanshan University, Qinhuangdao 066004, China; han0325@stumail.ysu.edu.cn (H.Z.); d202210477@xs.ustb.edu.cn (K.L.); ljzhou@stumail.ysu.edu.cn (L.Z.); ttz@stumail.ysu.edu.cn (T.Z.); hujie@ysu.edu.cn (J.H.)

[2] State Key Laboratory of Metastable Materials Science and Technology, Yanshan University,
Qinhuangdao 066004, China

[3] Department of Environmental Engineering, Hebei University of Environmental Engineering, Qinhuangdao 066102, China; jixianbing@hebuee.edu.cn (X.J.); cuiyanshuai@hebuee.edu.cn (Y.C.)

[4] Centre for cancer immunology, Faculty of medicine, University of Southampton,
Southampton SO16 6YD, UK; gm1c21@soton.ac.uk

* Correspondence: hanzs@ysu.edu.cn (Z.H.); lgwang@ysu.edu.cn (L.W.)

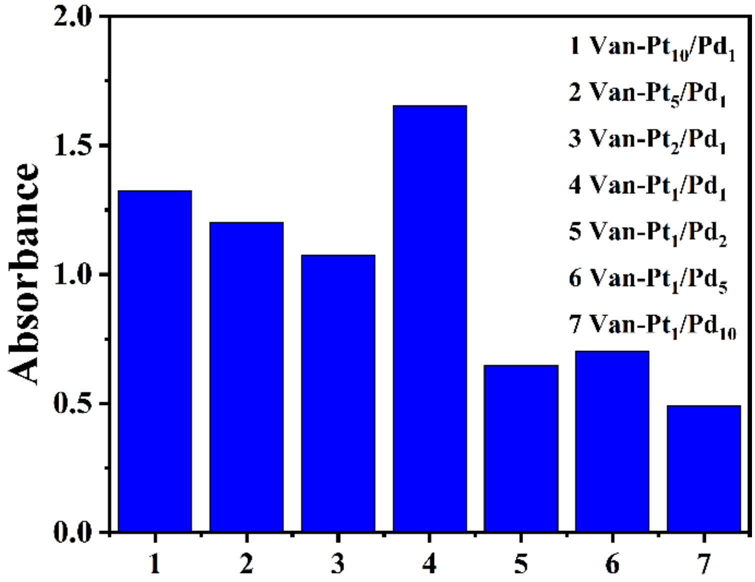

Figure S1. Oxidase-like activity of Van-Pt$_m$/Pd$_n$ NPs with different ratios of Pt and Pd at 652 nm.

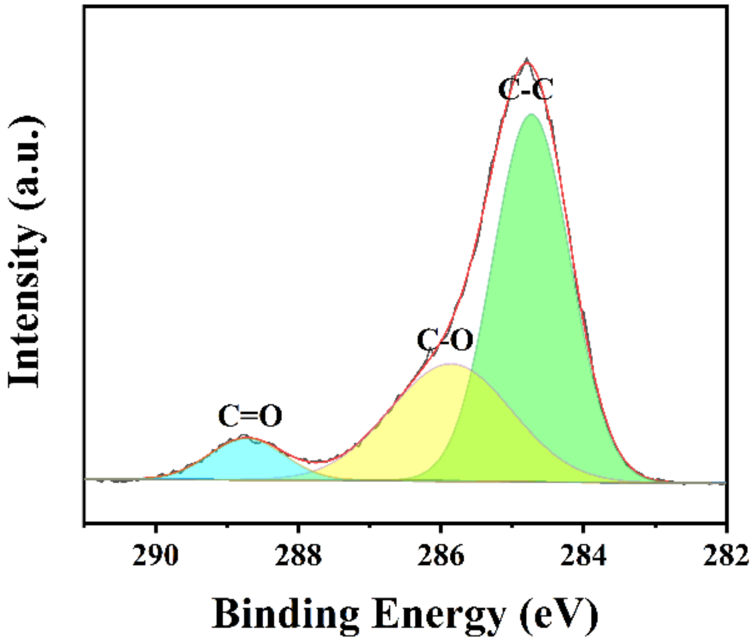

Figure S2. High resolution XPS spectrum of C.

As shown in Figure S3, infrared spectra of Van and Van-Pt_1/Pd_1 NPs were determined by Fourier transform infrared spectrometer. There are four distinct peaks at 3256 cm^{-1}, 3063 cm^{-1}, 1661 cm^{-1} and 1060 cm^{-1}, respectively. Among them, 3256 cm^{-1} and 3036 cm^{-1} are caused by O-H stretching vibration and C-H stretching vibration on the benzene ring, respectively. The characteristic peaks of 1060 cm^{-1} and 1661 cm^{-1} are R-O-R and R-CO-NH_2 in the free Van spectrum, respectively[1]. The infrared spectra of

Van and Van-Pt_1/Pd_1 NPs showed consistency in peak positions, which indicated that the Pt_1/Pd_1 NPs were attached to the Van and had a stable structure.

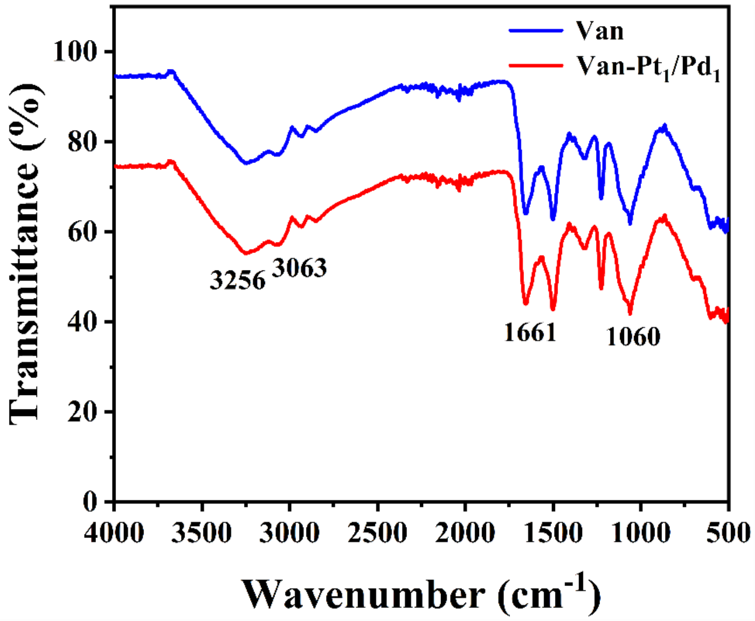

Figure S3. Infrared spectra of Van and Van-Pt_1/Pd_1 NPs

References

1. Abdelaziz, M. M.; Hefnawy, A.; Anter, A.; Abdellatif, M. M.; Khalil, M. A. F.; Khalil, I. A., Respirable spray dried vancomycin coated magnetic nanoparticles for localized lung delivery. *Int. J. Pharm.* **2022**, *611*, 121318.
